# Effects of the Ketogenic Diet on Muscle Hypertrophy in Resistance-Trained Men and Women: A Systematic Review and Meta-Analysis

**DOI:** 10.3390/ijerph191912629

**Published:** 2022-10-03

**Authors:** Salvador Vargas-Molina, José L. Gómez-Urquiza, Jerónimo García-Romero, Javier Benítez-Porres

**Affiliations:** 1 Department of Physical Education and Sport, Faculty of Sport Sciences, EADE-University of Wales Trinity Saint David, 29018 Málaga, Spain; 2 Physical Education and Sports Area, Faculty of Medicine, University of Málaga, 29010 Málaga, Spain; 3 Department of Nursing, Faculty of Health Sciences, University of Granada, 18071 Granada, Spain

**Keywords:** ketosis, strength, muscle mass, muscle protein synthesis, body composition, body building

## Abstract

Reviews focused on the ketogenic diet (KD) based on the increase in fat-free mass (FFM) have been carried out with pathological populations or, failing that, without population differentiation. The aim of this review and meta-analysis was to verify whether a ketogenic diet without programmed energy restriction generates increases in fat-free mass (FFM) in resistance-trained participants. We evaluated the effect of the ketogenic diet, in conjunction with resistance training, on fat-free mass in trained participants. Boolean algorithms from various databases (PubMed, Scopus. and Web of Science) were used, and a total of five studies were located that related to both ketogenic diets and resistance-trained participants. In all, 111 athletes or resistance-trained participants (87 male and 24 female) were evaluated in the studies analyzed. We found no significant differences between groups in the FFM variables, and more research is needed to perform studies with similar ketogenic diets and control diet interventions. Ketogenic diets, taking into account the possible side effects, can be an alternative for increasing muscle mass as long as energy surplus is generated; however, their application for eight weeks or more without interruption does not seem to be the best option due to the satiety and lack of adherence generated.

## 1. Introduction

Ketogenic diets (KDs) require the drastic reduction in carbohydrates (CHO), specifically to less than 50 g per day or 10% of total caloric intake, accompanied by a substantial increase in fat, and, in some cases, protein [1]. KDs have been proposed as alternatives under three general objectives: increases in athletic performance [2,3,4]; improvement of different pathologies [5], including evaluation of healthy parameters [6]; or improvements in body composition [7,8].

Research targeting body composition has focused for decades on obese patients [9], or those of normal weight but not trained [10,11]. The application of this nutritional strategy in resistance-trained participants began recently, with data obtained from our laboratory [12] and the investigations of Greene et al. [13] and Kephart et al. [14]. As a result of this, more studies have emerged that expand the evidence related to KDs and resistance-trained participants.

Additionally, research related to body composition is based on two main parameters: the reduction in adipose tissue, evaluated by fat mass (FM); and the increase in muscle hypertrophy, where fat-free mass (FFM) and muscle thickness are determined by ultrasound.

Hypertrophy is defined as an increase in muscle mass and volume. During this process, the contractile elements enlarge and the extracellular matrix expands [15]. To achieve this, it is necessary to manipulate the diet and the programming variables in strength training. Hypertrophy-targeted training focuses on the manipulation of programming variables where volume is the main variable [16], and where sets should be performed to or near failure (0–4 reps in the chamber) [17]. A wide range of repetitions, beyond 6–12, generates hypertrophy [18,19].

In this review, we will focus on increasing FFM. With regard to the increase in muscle tissue/FFM, the current evidence on nutritional strategy focuses on two basic parameters: a) generate an energy surplus; and b) consume between 1.6 and 2.2 g·kg^−1^ BM·d^−1^ protein [20]. It is reported that a smaller surplus is required in resistance-trained athletes (+5–10% above maintenance) and more in sedentary individuals and resistance-training novices (+10–20% above maintenance), to optimize protein synthesis, minimizing the accumulation of adipose tissue [20]. However, for the FM reduction phase, it is necessary to generate an energy deficit, in which the protein dose should be increased to between 1.8 and 2.7 g·kg^−1^ BM·d^−1^ [21] or even 2.3 and 3.1 g·kg^−1^ BM·d^−1^ [22] so as not to generate loss in muscle tissue. CHOs have traditionally been used as the main energy source in strength training, with levels of between 3 and 7 g·kg^−1^ BM·d^−1^ [23]. To increase muscle mass, in the off season-phase the levels recommended are between 4 and 7 g·kg^−1^ BM·d^−1^ [24], with a lipid intake between 0.8 and 1.3 g·kg^−1^ BM·d^−1^ [24]. The incorporation of KDs in the athletic and aesthetic environment, based on body composition, has now led to an alternative to generate this surplus, through the drastic reduction in CHOs and a concomitant increase in fat. For this reason, we conducted a systematic review of the evidence from randomized controlled trials (RCTs) investigating the efficacy of KDs on parameters related to muscle hypertrophy in resistance-trained participants.

## 2. Materials and Methods

This review and meta-analysis were carried out based on the guidelines proposed by (PRISMA) [25]. We describe the methodology used to systematically present our findings on the effects of KDs on body composition in strength-trained participants. The protocol for this systematic review was registered in protocolos.io (DOI: https://doi.org/10.17504/protocols.io.4r3l27m9qg1y/v1 (accessed on 31 August 2022).

### 2.1. Search Strategy

We systematically searched online medical databases, including Web of Science, PubMed/Medline, and SCOPUS for entries up to March 2022. The following search terms were used: (“ketogenic diet” OR “ketogenic dieting” OR “low carbohydrate diet” OR “low carbohydrate ketogenic diet” OR “very low carbohydrate diet”) AND (“body composition” OR “fat-free mass” OR “lean body mass” OR “LBM” OR “FFM” OR “ultrasound”) AND (“resistance training” OR “strength training” OR “muscle” OR “muscle mass” OR “hypertrophy” OR “training hypertrophy” OR “trained men” OR “training males” OR “trained women”).

### 2.2. Study Selection

Titles and abstracts of all articles obtained from the initial search were individually reviewed by two authors (S.V.M and J.B.P). The inclusion criteria for the articles were: (a) randomized trials, with a minimum duration of eight weeks; (b) the use of a KD in resistance-trained participants, competitors or elite athletes; (c) evaluation of body composition by means of dual-energy X-ray absorptiometry (DXA), bioimpedance or ultrasound; (d) data presented as means and standard deviations; (e) no intervention using nutritional or dietary supplements; and (f) the text was in English and available in full. Exclusion criteria included: (a) research conducted on animals; (b) systematic reviews or meta-analyses or uncontrolled experimental studies; (c) research without a control group; (d) joint interventions in the KD group; and (e) research carried out with anthropometry.

### 2.3. Data Extraction

From each selected article, information was extracted including the name of the first author, the date of publication, the average age of the participants, and the gender of both the experimental and control groups, in addition to the study design, the participants, the duration, the composition of the diet, and the means and standard deviations (SD) of both groups. Data related to body weight and FFM were selected.

Additionally, any disagreement in the selection of material to be included by the two researchers (S.V.M and J.B.P) was resolved by a third researcher.

### 2.4. Quality Assessment

Risk of bias assessment was conducted using the Cochrane method [26]. The elements evaluated were the following: random sequence generation, allocation concealment, blinding of participants and personnel, blinding of outcome assessment, incomplete outcome data, selective reporting, and other bias.

Studies were classified as high risk of bias, low risk of bias, or unclear bias, for each item assessed, based on the recommendations of the Cochrane Handbook.

RevMan Web software (London, UK) was used to develop meta-analyses. Meta-analyses were performed assessing the difference in post-intervention means and were performed with fixed effects if the heterogeneity value (I2) was less than 50%. Publication bias was assessed using funnel plots.

## 3. Results

**Figure 1:**
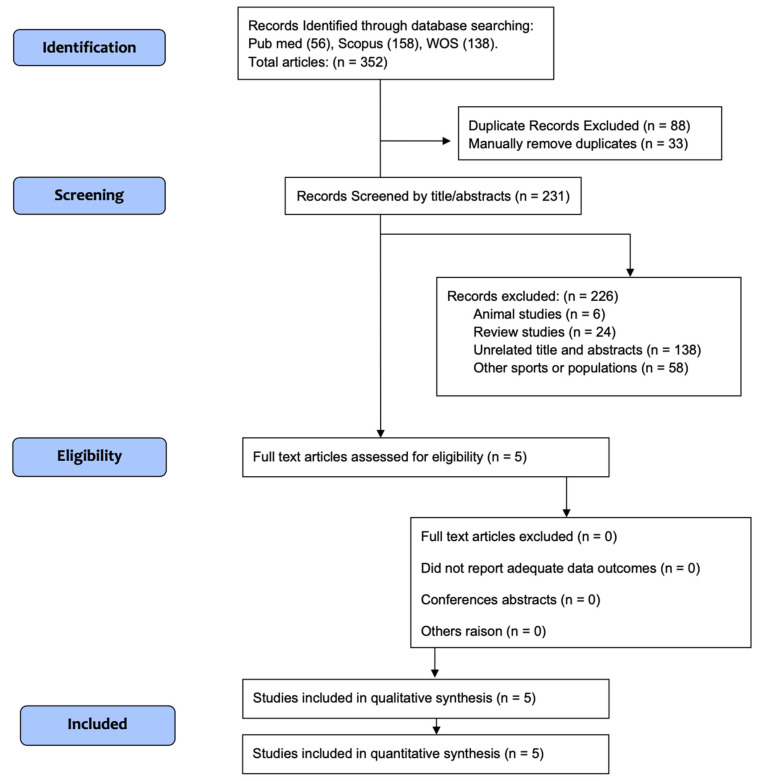
CONSORT Diagram.

The flow of studies in our meta-analysis is depicted in Figure 1, from 352 possibly relevant references. Of all the investigations, one did not present a complete text, so it was discarded. References were reviewed to determine eligibility. Finally, only five investigations met the eligibility criteria. These studies included 48 participants consuming a KD diet and 53 in control groups.

The characteristics of these five randomized controlled trials are presented in Table 1. Of these studies, four were evaluated by DXA, and one by BIA [27]. In addition, one also presented ultrasound results [28]; however, the FFM data were not provided. Only one investigation did not report nutritional data, although it used an ad libitum strategy [14]. In addition, all the investigations lasted between 8 and 12 weeks; however, ref [13] a crossover study was carried out, applying 12 weeks of a KD diet, 3 weeks of wash-out, and another 12 weeks of a No-KD protocol.

None of the variables analyzed showed the existence of publication bias, and heterogeneity was 0% in all analyses.

**Figure 2:**
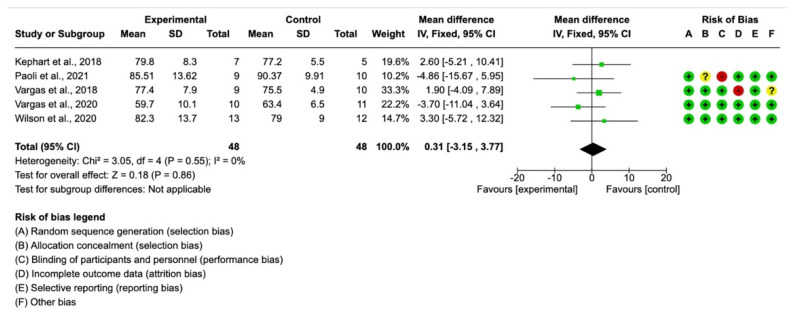
Forest plot for weight [12,14,27,28,29].

In the total mass variable, five studies provided the necessary data, with a final sample of *n* = 48 participants in the intervention group and *n* = 48 in the control group. The mean difference with a 95% confidence interval was 0.31 (−3.15, 3.77) in favor of the control group, although the differences were not significant (*p >* 0.05), as shown in Figure 2.

**Figure 3:**
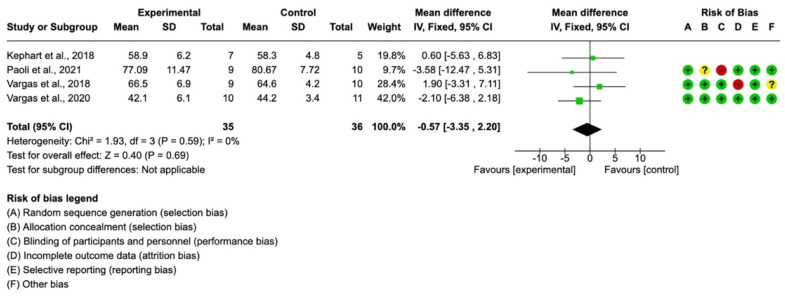
Forest plot for fat-free mass [12,14,27,29].

With regard to the FFM variable, four studies were included, with a final sample of *n* = 35 participants in the intervention group and *n* = 36 in the control group. The mean difference with a 95% confidence interval was −0.57 (−3.35, 2.20) in favor of the experimental group, although the differences were not significant. (*p* > 0.05), as shown in Figure 3.

## 4. Discussion

The results of our meta-analysis did not show significant differences between the control group and the experimental group in the FFM variable, although the differences between means were favorable to the experimental group. However, the meta-analysis of Koerich et al. [30] showed better results for FFM for the non-ketogenic group. The difference between our work and previous studies is that it focuses on participants trained in strength, and the review of Koerich et al. [30] examined trained athletes from different sports modalities.

Other reviews reported increases in FFM over 386 participants, although they included different populations and protocols without RT training [31]. However, the review and meta-analysis of Ashtary-Larky et al. [8], which included 13 studies with a total of 244 participants undertaking RT training and KD diets, found significant reductions in FFM in the KD group. The loss of FFM in the meta-analysis of Ashtary-Larky [8] may be due to the fact that, of the thirteen studies evaluated, eight were carried out ad libitum [13,14,29,32,33,34,35,36], and one of them did not report the nutritional data [37]. Therefore, only four studies included specific prescriptions for the consumption of total calories [12,27,28,38]. However, while Vargas et al. [12] initially prescribed ≈39 kcal·kg^−1^·d^−1^, there was no nutritional record; therefore, we cannot say that the participants consumed the total energy required. The same difficulty applies to the study by Rhyu et al. [38] where experimental records are unavailable. In research carried out in our laboratory [29], we checked the difference between the initial nutritional prescription and the actual record. Where the prescribed intake was ≈45 kcal·kg^−1^·d^−1,^ at the end of the eight weeks, the reported mean was ≈40 kcal·kg^−1^·d^−1^, unlike the group that did not engage in a KD, who reached the prescribed calorie intake at the beginning of the study. Therefore, and given that energy surplus is currently considered the main factor in increasing muscle mass [20], we must consider the main limitation to achieving it: the satiety generated by KD diets [39,40]. In fact, ad libitum KDs have shown a lower energy consumption [40], indicating it is difficult to reach an adequate intake of calories, and thus impairing adherence to the diet. The satiating effect of KDs that do not produce an increase in FFM can optimize the reduction in fat mass [8,31]. However, when total calories are equalized, there is no additional advantage in KDs [41]. 

Only the investigation of Wilson et al. [28] carried out a reintroduction of carbohydrates in the post-experiment evaluation. As DXA evaluates FM and FFM, and the accumulation of muscle glycogen is accompanied by approximately 3 g of water, carbohydrate loading will increase the intracellular content, FFM values will be higher, and the differences in the stored glycogen and associated water content will influence both total mass and FFM [42]. This limitation must be considered before concluding that KDs do not cause an increase in FFM, especially when accompanied by RT.

However, no research has corrected for fat-free adipose tissue (FFAT). In this regard, as the amount of adipose tissue is reduced, so will FFM, since fat-free adipose tissue is included in the DXA measurement of fat-free tissue mass [43]. In fact, considering the model proposed by Heymsfield et al. [44] to reduce the difference between the FFM component at the molecular level and the corresponding value measured at the macro level in the tissue/organ (e.g., MRI or DXA), it is assumed that 85% of adipose tissue is fat and the remaining 15% is fat-free adipose tissue (FFAT). If the participants in our study had similar increases in FFM, these data would not be entirely accurate.

It should be noted that the study by Gregory et al. [35] was excluded because, despite being Crossfit^®^ athletes, the inclusion criteria specified only that the sample should be between 18 and 60 years old and have completed a minimum of one month of active training, which could not be considered advanced.

Although our results are not favorable for an increase in FFM with the use of a KD, it does not mean that the cross-section of skeletal muscle cannot be increased. In fact, as mentioned, DXA evaluates FM and FFM; therefore, it is not a direct indicator of muscle hypertrophy. Ultrasound evaluation has proven to be a more reliable marker for this purpose [45]. 

For this reason, the investigation of Wilson et al. [28] used ultrasound, in addition to DXA, finding favorable effects of a KD. This was contrary to the findings of Kephard et al. [14] of a decrease in muscle thickness in the legs, although that study involved the previously mentioned limitations in nutritional control.

Additionally, training focused on muscle mass gain is based on volume as the main programming variable [16], and is optimized with weekly volumes of between 16 and 24 series [46], where it can be more efficient to distribute it in frequency 2 [47]. 

An excess of series per session, in advanced participants, can lead to *wasted sets*, where protein synthesis is not optimized [48]. For this reason, an average volume would be between 8 and 12 sets/session, with up to 20 total sets per week recommended to reach the maximum recoverable volume [49]. In this way, the review in [50] found that carbohydrate consumption could improve strength performance in training volumes greater than 10 sets per session. Since our goal was muscle hypertrophy in advanced athletes, it does not appear that carbohydrate restriction would affect the set volume needed per session in advanced participants.

This study has some limitations. The interventions using KDs included in the meta-analysis were not exactly similar and the number of studies included in the meta-analysis was low. Thus, the results should be treated with caution and future studies should perform a protocolized and similar KD intervention.

Additionally, the use of KDs has possible side effects that must be considered in study populations. Cases of skin rash and “keto flu” have been reported, where nausea, tremors, lack of energy, vomiting, fainting, and halitosis can occur [51]. As a general rule, in non-pathological and trained subjects, as is the case in our review, the metabolic symptoms associated with the change in energy from carbohydrates to fats usually disappear in a few days.

We must emphasize that the five selected studies met the protein ranges established for the increase in myofibril proteins (1.6 and 2.2 g kg BM·d^−1^ of protein) [20]. In addition, we should consider Leucine as the main amino acid in protein synthesis [52,53], being found in greater quantity in the foods chosen in these investigations (dairy, meat, fish, and eggs). However, consuming more protein, and thus more Leucine per meal, will not increase myofibrillar protein synthesis [54]. None of the selected studies established plant-based diets. For these reasons, we did not find an advantage in any selected study based on a slightly higher protein intake.

## 5. Conclusions

We conclude that by applying a KD diet without energy restriction, significant increases in FFM can be achieved, since the total energy prevails for this objective. However, due to the satiating effect and the consequent lack of adherence that a KD generates, it does not seem to be an optimal nutritional strategy, especially if it is maintained continuously for eight weeks or more.

## Figures and Tables

**Table 1:** Characteristics of five randomized controlled clinical trials.

Reference	Sample	Duration	Ketogenic Diet Intervention	Control Intervention	Main Results	Country	Measurement of FFM
Paoli et al. (2021) [27]	Body builder males (KD; *n* = 9, NKD; *n* = 10) 27.42 ± 10.54. years BMI; 26.80 ± 1.91 kg/m^2^	8 weeks	45 kcal∙kg-FFM^−1^∙d^−1^ Distribution 2.5 g∙kg^−1^·d^−1^ PRO, <50 g∙d^−1^ CHO, remaining calories FAT;	45 kcal∙kg-FFM^−1^∙d^−1^ Distribution: 2.5 g∙kg^−1^·d^−1^ PRO, 55% CHO, remaining calories FAT)	No significant changes in FFM in KD (>0.05). Significant changes in NKD (<0.05).	Italy	BIA
Vargas-Molina et al. (2020) [29]	Resistance-trained women (*n* = 21) 27.6 ± 4. years 62.3 ± 7.8 kg 162 ± 6.6 cm	8 weeks	40–45 kcal∙kg-FFM^−1^∙d^−1^ Distribution: 1.7 g∙kg^−1^·d^−1^ PRO, 30–40 g∙kg∙d^−1^ CHO, remaining calories FAT.	40–45 kcal∙kg-FFM^−1^∙d^−1^ Distribution: 1.7 g∙kg^−1^·d^−1^ PRO, 1 g∙kg^−1^·d^−1^ FAT, remaining calories CHO	No significant changes in FFM in KD (−0.7 ± 1.7 kg; *p* = 0.202; d = −0.1)	Spain	DXA
Wilson et al. (2020) [28]	Resistance-trained males. (*n* = 25) KD: 23.0 ± 4.5 and NKD: 21.3 ± 3.7 years	11 weeks	Distribution: 5% CHO 20% PRO 75% FAT;	Distribution: 55% CHO, 20% PRO, 25% FAT	FFM increased (2.4% and 4.4%; KD and WD) at week 10. FFM, only increased KD (4.8%) between weeks 10 and 11.	United States	DXA Ultrasonography
Vargas et al. (2018) [12]	Resistance-trained males, (*n* = 24) 30 ± 4.7 years; 76.7 ± 8.2 kg 174.16 ± 7 cm	8 weeks	39 kcal∙kg-FFM^−1^∙d^−1^ Distribution: <10% CHO, 20% PRO, 70% FAT	39 kcal∙kg-FFM^−1^∙d^−1^ Distribution: 55% CHO, 20% PRO, 25% FAT	FFM in KD (*p* > 0.05). No increase. NKD (*p* < 0.05) showed increased FFM.	Spain	DXA
Kephart et al. (2018) [14]	Resistance-trained males/women (*n* = 12; 9 M, 3 W) 31 ± 2 years; 80.3 ± 5.1 kg	12 weeks	Not reported Ad libitum	Not reported Ad libitum	FFM no significant changes between groups. Leg FFM decreased in KD (1.4%-*p* = 0.068),	United States	DXA

## Data Availability

Data will be made available upon reasonable request to the corresponding author.
